# Supplementary material for: At the Crossroads of the cGAS-cGAMP-STING Pathway and the DNA Damage Response: Implications for Cancer Progression and Treatment
Source: Pharmaceuticals (Basel). 2023 Dec 1;16(12):1675. doi: 10.3390/ph16121675 (PMC10747911; doi:10.3390/ph16121675)
Supplement: Supplementary file 1 [file pharmaceuticals-16-01675-s001.zip › pharmaceuticals-2667839-supplementary.pdf]

Supplementary Table S1. Genes/proteins that modulate the function of cGAS-cGAMP-STINg axis in oncology

Gene / Protein name	Human uniprot	Protein MW (kDa)	Dominant location	Natural cofactors, activators, etc.	Inhibitors, drugs, etc.	Cell / issue Expression	Activity / modulation of STING	General Function	Role in oncology	Other diseases	Most famous protein interactors
STING1 STING	Q86WV6	42	ER→G with cGAS	cGAS	STING inhibitor C-178, STING agonist-1 (G10)	UB	assembly	dsDNA and dsRNA response	dual	SAVI	TBK1, IFI16, IRF3
CGAS	Q8N884	59	C, N	dsDNA	G150, PF-06928215, RU.521	UB	cGAMP synthase	ds DNA sensing	dual	-	-
PARP1	P09874	113	N		Veliparib (ABT-888), Olaparib (AZD2281)	UB	poly(ADP-ribose) polymerase	DDR	As a cancer treatment, PARP inhibitors stop the PARP from doing its repair work in cancer cells and the cells dies	-	PARP1
PARP2	Q9UGN5	66	N		Veliparib (ABT-888), Olaparib (AZD2281)	UB	-	DDR		-	-
PARP9	Q8IXQ6	96	C→N upon IFNG or IFNB1		-	UB	-	DDR (recruits E3 ligase DTX3L)		-	PARP1, STAT1
TFAM	Q00059	29	M		-	UB	activates STING	DNA transcription and replication initiation	-	MTDPS15	-
TBK1	Q9UHD2	84	C		TBK1/IKKε-IN-2, TBK1/IKKε-IN-5	UB	phosphorylating innate adapter proteins MAVS, STING1	IRF3 activator	TBK1 promotes the proliferation and survival of cancer cells by activating intrinsic signaling within cancer cells	GLC1P, FTDALS4, IIAE8	IRF3, OPTN, TRAF3
TREX1 DNase III intronless	Q9NSU2	33	ER→N upon ROS	Mg		UB	3'-exonuclease for with mismatched 3' termini	DDR,	TREX1 controls dsDNA sensing in tumor and immune cells by	AGS1, SLE, CHBL1	SET complex (ANP32A, APEX1,

								degradation of IF-stim DNA	preventing aberrant dsDNA buildup that triggers STING-mediated Type 1 Interferon (IFN) induction leading to priming of the adaptive immune system		HMGB2, NME1, SET and TREX)
HMGB1	P09429	25	N	-	Glycyrrhizin (NSC 167409), Ammonium Glycyrrhizinate	UB	Activation by oligomerization of STING	Binds DNA (transcriptional control), binds TLR4 for inflammation	DAMP	-	HMGB2, PARP1
FABP5	Q01469	15	C	-	SBFI-26	Mostly Squamous epithelial cells	?	Binds amantadine and free ARA and RA	Promotes cancers through lipid metabolism	-	-
PPP6C	O00743	35	C	Mn		UB	inactivates cGAS and STING1	Catalytic PP6 (S/T): decelerates mitosis, activates RIG1	dual	-	-
ENPP1	P22413	105	C	Zn	Enpp-1-IN-1	Mostly Glandular and Specialized epithelial cells, Mesenchymal cells	?	Hydrolyzes ATP to generate AMP and inorganic pyrophosphate (PPi), thereby inhibiting mineralization processes.	ENPP1 expression is heightened in M2 macrophages in the presence of cancer and promotes tumor growth and spread	OPLL, GACI1, T2D, ARHR2, COLED)	-
ATM	Q13315	351	C, N	DNA double-strand breaks (DSBs)	KU-55933, CP-466722, Wortmannin	UB	activates STING	DNA damage sensor	-	AT	TP53, BRCA1
IFI16	Q16666	88	N	dsDNA	-	UB	activates STING	DNA binding	IFI16 promotes cervical cancer progression by upregulating PD-L1 in immunomicroenvironment through STING-TBK1-NF-kB pathway	-	TMEM173, BRCA1

ZBP1	Q9H171	46	C		-	Blood & immune cells	activates STING	Binds Z-RNA; essential mediator of pyroptosis, necroptosis and apoptosis	ZBP1 as the key regulator of tumor necroptosis and provides a potential drug target for controlling tumor metastasis	-	-
DDX41	Q9UJV9	70	N	-	-	UB	cross-interaction with the cGAS pathway through the STING	Recognize the bacterial second messengers cyclic di-GMP and cyclic di-AMP, resulting in the induction of genes involved in the innate immune response	-	MPLPF	-
RAD50	Q92878	154	N	Zn	Mirin	Mostly Neuronal cells, Glial cells	cross-interaction with the sGAS pathway through the STING	double-strand break (DSB) repair	Downregulation of RAD50 sensitized colorectal cancer cells to irradiation with reduction in DSB repair efficiency	NBSLD	MRE11A, NBN, BRCA1
RAD51	Q06609	37	C, N, M	-	RAD51 inh.1, Amuvatinib (MP-470)	Mostly Germ cells, Trophoblast cells	-	DNA repair through homologous recombination (HR)	Rad51 also participates in the tumor metabolism, metastasis and chemotherapeutic resistance	BC, MRMV2, FANCR	BRCA2, PALB2, BRCA1
MRE11A	P49959	81	N	Mn	PFM01, Mirin	UB	cross-interaction with the sGAS pathway through the STING	double-strand break (DSB) repair	Marker for targeted radiosensitization strategies	ATLD1	NBN, RAD50, MDC1, BRCA1
MYD88	Q99836	33	C, N	-	TJ-M2010-5, ST 2825, Schaftoside	Mostly Blood & immune cells	can form a complex with STING	Activates IRF1, NF-kB	In human cancers, MyD88 protein acts as a bridge between the inflammatory signaling from the TLR/IL-1R and Ras oncogenic signaling pathway	IMD68, WM1	IRAK1

IRF3	Q14653	47	C, N	-	C6orf106	UB	product of the cGAS-cGAMP-STING-TBK1 pathway	Key transcriptional regulator of type I interferon (IFN)-dependent immune responses which plays a critical role in the innate immune response against DNA and RNA viruses	Tumor suppressor and a prognosis marker of the CRC patients with an unexpected mechanism	IIAE7	IRF3, CREBBP, TBK1
IRF7	Q92985	54	C, N	-	-	Mostly Blood & immune cells	product of the cGAS-cGAMP-STING-TBK1 pathway	Key transcriptional regulator of type I interferon (IFN)-dependent immune responses and plays a critical role in the innate immune response against DNA and RNA viruses	Act as a tumor suppressor that inhibits the proliferation and metastasis of cancer cells	IMD39	-
RIG-I	O95786	107	C	Zn	RIG012	Mostly Glial cells	?	Innate immune receptor that senses cytoplasmic viral nucleic acids and activates a downstream signaling cascade leading to the production of type I interferons and pro-inflammatory cytokines	Activation of RIG-I in natural killer (NK) cells leads to the secretion of TRAIL, which induces cancer cell death	SGMRT2	TRIM25, MAVS
MAVS	Q7Z434	57	M	-	-	UB	?	Coordinate pathways leading to the activation of NF-kappa-B, IRF3 and IRF7	MAVS influences biological behavior by regulating the immune response and that it can be used as a predictive marker for poor prognosis in ovarian cancer	-	TRAF3, RIG-I
RIPK3	Q9Y572	57	C, N	Activity is stimulated by ZBP1, which senses double-stranded Z-RNA structures	-	UB	Vice versa, necroptotic signaling stimulates STING, whereas suppression of RIPK3 inhib-its STING by redirecting	Activates necroptosis and apoptosis	Similar to apoptosis, RIPK3-signaling mediated necroptosis is inactivated in most types of cancers, suggesting RIPK3 might play a critical suppressive	-	RIPK1

							STING to autophagosomes. In the mouse, STING agonists induce necroptosis in primary macrophages in vitro in a STING and RIPK3-dependent mode linked to production of and IFN and TNF		role in the pathogenesis of cancers. However, in some inflammatory types of cancers, such as pancreatic cancers and colorectal cancers, RIPK3 signaling might promote cancer development by stimulating proliferation signaling in tumor cells and inducing an immunosuppressive response in the tumor environment		
NLRP3	Q96P20	118	C, ER, G	Extracellular ATP, nigericin, reactive oxygen species, crystals of monosodium urate or cholesterol, amyloid-beta fibers, environmental or industrial particles and nanoparticles	MCC950 Sodium, INF39, NLRP3 Inflammasome Inhibitor I	Mostly Blood & immune cells	Pyroptosis, the cell death pathway that employs the NLRP3 inflammasome, and ly-sosomal cell death are promoted by STING after its translocation to the lysosome through an increase in lysosomal membrane permeabilizaton, especially in human myeloid cells we may speak about the major cGAS-STING-NLRP3 pathway that works as a default death program in these cells	Activation in response to defects in membrane integrity, leading to secretion of inflammatory cytokines IL1B and IL18 and pyroptosis	NLRP3 in the primary lesion of cancer cells drives the production of pro-IL-1β, DC maturation, and the secretion of IL-1β to support the evolution of tumor-specific CD8+ T cells	MWS, CINCA, KEFH	PYCARD
NCOA4	Q13772	70	N	-	-	UB	STING-associated followed by ferropoptosis	Enhances the androgen receptor transcriptional activity in prostate cancer cells	In cancer cells, NCOA4 is a critical regulator of ferroptosis, and mutations in NCOA4 can lead to decreased ferroptosis of	-	AR

								tumor cells by reducing intracellular free iron accumulation, glutathione synthesis, and reactive oxygen species, among other effects (ROS)		
SRC	P12931	60	C	-	Src Inhibitor 1, Saracatinib, Bosutinib and etc.	UB	SRC restricts cGAS signaling in human cell lines through SRC small molecule inhibitors, depletion, and overexpression. cGAS and SRC interact in cells and in vitro, while SRC directly inhibits cGAS enzymatic activity and DNA binding in a kinase-dependent manner	Src is a non-receptor protein tyrosine kinase that transduces signals that are involved in the control of a variety of cellular processes such as proliferation, differentiation, motility, and adhesion	The proto-oncogene c-Src (Src) is a nonreceptor tyrosine kinase whose expression and activity is correlated with advanced malignancy and poor prognosis in a variety of human cancers.	THC6	EGFR
EIF2AK3 (PERK)	Q9NZJ5	125	ER	-	-	UB	STING–PKR-like endoplasmic reticulum kinase (PERK)–eIF2α pathway, a cGAS–STING mechanism, enabling an innate immunity control of cap-dependent messenger RNA translation	Phosphorylates the alpha subunit of eukaryotic translation initiation factor 2 (EIF2S1/eIF-2-alpha) in response to various stress conditions	The high expression of PERK could promote the infiltration of multiple immune cells in the tumor microenvironment and could deteriorate the outcomes of patients with breast and thyroid cancers, suggesting that PERK as well as tumor-infiltrating immune cells could be taken as potential biomarkers of prognosis	WRS	-
EIF2S1 (eIF2α)	P05198	36	C	Activity is regulated by phosphorylation at Ser-49 and Ser-52, which	Salubrinal	UB		Member of the eIF2 complex that functions in the early steps of protein synthesis by forming a	-	-	-

				stabilizes the eIF2/GDP/eIF-2B complex and prevents the eIF-2B-mediated exchange of GDP for GTP				ternary complex with GTP and initiator tRNA			
SLC19A1	P41440	65	CM	-	-	Mostly Trophoblast cells	Importer of cyclic GMP-AMP dinucleotides	Importer of immunoreactive cyclic dinucleotides, such as cyclic GMP-AMP (2'-3'-cGAMP, thus playing a role in triggering larger immune responses	-	MEGAF	-
SLC46A2	Q9BY10	51	CM, EM	-	-	Mostly Specialized epithelial cells	Dominant importer of cyclic GMP-AMP dinucleotides (cGAMPs) in monocyte and macrophage cell lineages	Selectively imports cGAMPs derived from pathogenic bacteria such as 3'3'-cGAMP thus providing for differential immune recognition of pathogenic versus commensal bacteria	During tumorigenesis may transport extracellular tumor-derived 2'3'-cGAMP across the plasma membrane of M1-polarized macrophages to activate the anti-tumoral stimulator of interferon genes (STING) pathway	-	-
LRRC8A (SWELL1)	Q8IWT6	94	CM, LM	-	-	UB	Complexes containing LRRC8D inhibit transport of 2'-3'-cGAMP	Essential component of the volume-regulated anion channel (VRAC), an anion channel required to maintain a constant cell volume in response to extracellular or intracellular osmotic changes	LRRC8A Is a Prognostic Biomarker and Therapeutic Target for Pancreatic Adenocarcinoma	AGM5	-
STAT3	P40763	88	C, N	-	Cryptotanshinone, Niclosamide, Stattic and etc.	UB	cGAS–STING cytosolic DNA sensing pathway is suppressed by JAK2-STAT3 in tumor cells	Through its regulation of gene activity, the STAT3 protein is involved in many cellular functions. It helps control cell growth and	Activated STAT3 in cancer cells induces the release of factors that block APCs maturation/activation and	HIES1, ADMIO1	EGFR

								division (proliferation), cell movement (migration), and the self-destruction of cells (apoptosis)	inhibits the generation of antigen-specific T cells, causing immune tolerance		
HELB	Q8NG08	123	C, N	Uses either magnesium or manganese ions to support helicase activity.	Inhibited by salt concentration greater than 100 mM.	Mostly Glial cells, Blood & immune cells	Damages to oncosuppressor genes, such as those in TP53BP1, MAD2L2, HELB, RIF1, is a prerequisite to carcino-genesis. The canonic cGAS-STING is involved in DDR pathway in the situation of loss/insufficiency of oncosuppressors such as BRCA1 or BRCA2	5'-3' DNA helicase involved in DNA damage response by acting as an inhibitor of DNA end resection	-	-	-
MAD2L2	Q9UI95	24	C, N	-	-	Mostly Germ cells		Adapter protein able to interact with different proteins and involved in different biological processes. During G1 and S phase of the cell cycle, the complex functions downstream of TP53BP1 to promote non-homologous end joining (NHEJ) and suppress DNA end resection	MAD2L2 has been suggested to serve as a tumor suppressor in several types of cancer, including colorectal cancer	FANCV	REV3L, REV1
TP53BP1	Q12888	214	N	-	UNC2170	UB		Double-strand break (DSB) repair protein involved in response to DNA damage, telomere dynamics and class-switch recombination (CSR) during antibody genesis	The tumor suppressor P53-binding protein 1 (53BP1) is a member of Tudor-containing proteins (including JMJD2 A, 53BP1, SGF29, Spindlin1, UHRF1, PHF1, PHF19 and SHH1) in 'reading' unique methylation events on histones in order to facilitate DNA damage repair or regulate transcription	-	TP53
RIF1	Q5UIP0	275	C,N	-	-	UB	?	Key regulator of TP53BP1 that plays a key role in the	RIF1 promotes cell growth and cancer stem	-	-

								repair of double-strand DNA breaks (DSBs) in response to DNA damage: acts by promoting non-homologous end joining (NHEJ)-mediated repair of DSBs	cell-like phenotype in NSCLC by activating the Wnt/β-catenin pathway		
BRCA1	P38398	208	C, N	The E3 ubiquitin-protein ligase activity is inhibited by phosphorylation by AURKA. Activity is increased by phosphatase treatment	BRCA1-IN-1	Mostly Germ cells	DNA repair defects due to BRCA1/2 mutation instigate immune signaling through the cGAS/STING pathway. The subsequent inflammatory signaling provides both tumor-suppressive as well as tumor-promoting traits	The BRCA1 protein has multiple functions in different cellular processes, including DNA repair, transcriptional activation, cell cycle regulation and chromatin remodeling	Mutations in the BRCA1 gene are associated with an increased risk of breast cancer in both men and women, as well as several other types of cancer	BC, BROVCA1, OC, PNCA4	BARD1, RAD51, TP53
BRCA2	P51587	384	C, N	-	CAM833	Mostly Germ cells, Blood & immune cells	BRCA2 deficiency instigates cGAS-mediated inflammatory signaling and confers sensitivity to tumor necrosis factor-alpha-mediated cytotoxicity	BRCA2 plays a role in transcriptional and cell cycle regulation, DNA repair, mitophagy and stabilization of replication fork	Breast cancer susceptibility gene 2 (BRCA2) is the main gene associated with hereditary breast cancers. However, a mutation in BRCA2 has also been found in other tumors, such as ovarian, pancreatic, thyroid, gastric, laryngeal, and prostate cancers	BC, PNCA2, BROVCA2, GLM3, MDB	RAD51, PALB2, BRCA1
TLR3	O15455	104	ER-membrane, EM	-	CU-CPT 4a (TLR3/dsRNA Complex Inhibitor)	Mostly Glial cells, Germ cells	?	Key component of innate and adaptive immunity. TLRs (Toll-like receptors) control host immune response against pathogens through recognition of	TLR3 stimulation with dsRNA is considered to directly promote tumor-cell apoptosis in many types of cancer, such as breast, melanoma,	IMD83	-

								molecular patterns specific to microorganisms. TLR3 is a nucleotide-sensing TLR which is activated by double-stranded RNA, a sign of viral infection. Acts via the adapter TRIF/TICAM1, leading to NF-kappa-B activation, IRF3 nuclear translocation, cytokine secretion and the inflammatory response	prostate, cervical, colon, and hepatocellular carcinoma. Poly (I:C), a TLR3 activator, directly causes apoptosis in cancer cells via a caspase-dependent pathway		
SETX	Q7Z333	303	C, N	-	-	UB	In cells with SETX and BRCA1 mutations, there is a noticeable accumulation of cytoplasmic RNA-DNA hybrids, products of R-loop processing. This accumulation leads to the activation of cGAS-STING.-	Senataxin plays an important cellular role at the interface of transcription and the DNA damage response and that the resolution of R-loop structures is a key event in the maintenance of genome stability	-	SCAN2, ALS4	BRCA1
HMMR (RHAMM)	O75330	84	C	-	-	Mostly Blood & immune cells, Germ cells, Trophoblast cells	Mouse models showed how HMMR magnifies the pro-oncogenic effects of mutated BRCA1. This leads to micronuclei formation and subsequent cGAS-STING activation. Both canonical and non-canonical NF-κB signaling pathways get activated as a result	When hyaluronan binds to HMMR, the phosphorylation of a number of proteins, including PTK2/FAK1 occurs. May also be involved in cellular transformation and metastasis formation, and in regulating extracellular-regulated kinase (ERK) activity	Hyaluronan-mediated motility receptor (HMMR) plays a pivotal role in cell proliferation in various cancers, including lung cancer. However, its function and biological mechanism in lung adenocarcinoma remain unclear	-	-

PALB2	Q86YC2	131	N	-	-	UB	?	Plays a critical role in homologous recombination repair (HRR) through its ability to recruit BRCA2 and RAD51 to DNA breaks	PALB2 is a tumor suppressor in which inherited mutations increase the risk of breast, ovarian, pancreatic, and other cancers. It plays a critical role in DNA repair and promotes antioxidant gene expression	BC, PNCA3	BRCA2, BRCA1, RAD51
FANCD2	Q9BXW9	164	N	-	-	Mostly Blood & immune cells, germ cells	-	Required for maintenance of chromosomal stability. Promotes accurate and efficient pairing of homologs during meiosis. Involved in the repair of DNA double-strand breaks, both by homologous recombination and single-strand annealing. May participate in S phase and G2 phase checkpoint activation upon DNA damage	-	FANCD2	FANCI, FANCL, BRCA2
MLH1	P40692	87	N	-	-	UB	Deficiency in mismatch repair (MMR), as seen in MLH1 deletion, also leads to accu-mulation of cytosolic DNA and activation of cGAS-STING, yielding interferon-β produc-tion	MLH1 is an MMR protein that forms a complex with DNA-repair protein PMS2, and coordinates the other DNA-repair protein effectors to repair mismatches arising during DNA replication	Tumor suppressor gene. Mutation in the MLH1 gene, this means you have a condition called Lynch syndrome. Lynch syndrome increases your risk for certain types of cancers, including: Colorectal (colon and rectal) cancer. Uterine (endometrial) cancer	ENDMC, CRC	PMS2, BLM, BRIP1
CHK1	O14757	54	C, N	Activated through phosphorylation predominantly	Schisandrin B	Mostly germ cells,	-	Serine/threonine-protein kinase which is required for checkpoint-mediated cell cycle arrest and activation	Phosphorylates multiple sites within the C-terminus of TP53, which promotes activation of	-	CDC25C, CLSPN

				by ATR but also by ATM in response to DNA damage or inhibition of DNA replication		glandular epithelial cells		of DNA repair in response to the presence of DNA damage or unreplicated DNA	TP53 by acetylation and promotes cell cycle arrest and suppression of cellular proliferation		
CHK2	O96017	61	N	Cofactor - Mg. Activated through phosphorylation at Thr-68 by ATM in response to DNA double-strand breaks. Activation is modulated by several mediators including MDC1 and TP53BP1	PV1019	UB	It is pertinent to note that the cGAS-STING pathway is intricately intertwined with numerous signaling pathways, including those involved in DNA damage, rendering it susceptible to varied modes of regulation. For instance, in the event of DNA damage, the activation of the ATM/Chk2 pathway induces a replication checkpoint within the cell	Checkpoint kinase 2 is a multifunctional enzyme whose functions are central to the induction of cell cycle arrest and apoptosis by DNA damage	Human genetic studies clearly show that CHEK2 is a multiorgan tumour susceptibility gene, but current evidence indicates that CHEK2 on its own does not predispose to cancer	LFS, PC, OSRC, BC	BRCA1, TP53
CXCL9	Q07325	14	Secreted	-		Blood & immune cells, glial cells	-	Cytokine that affects the growth, movement, or activation state of cells that participate in immune and inflammatory response	Attracts cells expressing the CXCR3 receptor including activated T and NK cells and has been shown to play a role in responses to immune checkpoint therapy	-	-
SOCS1	O15524	24	N	-	-	Blood & immune cells	-	Negative regulator of type I and type II interferon signaling, as well as that of other cytokines, including IL2, IL4, IL6 and leukemia inhibitory factor	SOCS1 is working as a negative-feedback regulator of cytokine signaling, a tumor suppressor and an oncogene	AISIMD	JAK2, IRF3

BLK	P51451	58	CM	Antibody-mediated surface engagement of the B-cell antigen receptor (BCR) which results in the phosphorylation of BLK on tyrosine residues, stimulates the enzymatic activity.	BLK-IN-2	Blood & immune cells	Phosphorylates CGAS, promoting retention of CGAS in the cytosol	Non-receptor tyrosine kinase involved in B-lymphocyte development, differentiation and signaling	B-lymphoid tyrosine kinase (Blk) is an oncogene and a potential target for therapy with dasatinib in cutaneous T-cell lymphoma (CTCL) Leukemia	MODY11	EGFR, STAT3
IDO1	P14902	45	C	Cofactor - heme b. Binds 1 heme group per subunit.	Linrodostat mesylate	Mostly glial cells, Glandular epithelial cells	-	Catalyzes the first and rate limiting step of the catabolism of the essential amino acid tryptophan along the kynurenine pathway.<br>Involved in the peripheral immune tolerance, contributing to maintain homeostasis by preventing autoimmunity or immunopathology that would result from uncontrolled and overreacting immune responses.	Acts as a suppressor of anti-tumor immunity	-	-
PD-1, PD-L1, CTLA4, LAG3	Q15116 Q9NZQ7 P16410 P18627	32 33 25 57	CM				cGAS-STING pathway is also important?	Immune checkpoint blockade relies on the binding of specific monoclonal antibodies to inhibitory immune proteins PD-1, PD-L1, CTLA4, LAG3	-		

ERCC1	P07992	33	C, N	-	-	UB	?	DNA repair and recombination	ERCC1 is a predictive biomarker for non-small cell lung cancer but Is antibody-dependent	COFS4	ERCC4, BRCA1
JAK2	O60674	131	C, N	Mg	Filgotinib, Abrocitinib, Ruxolitinib, Pacritinib and etc.	UB	For therapeutic cGAMP or other STING agonists, it might be necessary to block pathways JAK2-STAT3	Non-receptor tyrosine kinase involved in various processes such as cell growth, development, differentiation or histone modifications. Mediates essential signaling events in both innate and adaptive immunity.	-	BDCHS, PV	SOCS1, STAT3
GBP3	Q9H0R5	68	C, G	-	-	mostly epithelial cells	STING may increase resistance to temozolomide (TMZ) in pediatric high grade glioma (pHGG) through interaction with GBP3 (TMZ upregulates it), inducing expression of p62.<br>In patients, GBP3 expression is high and positively correlated with STING, NRF2, p62, and MGMT expression in human glioblastoma tumors	Interferon (IFN)-inducible GTPase that plays important roles in innate immunity against a diverse range of bacterial, viral and protozoan pathogens	-	-	-
TP53	P04637	44	N, C, ER	Zn	Roscovitine, Pifithrin-α hydrobromide, Nutlin-3, Pifithrin-α, p-Nitro, Ellipticine and etc.	UB	Within the DNA damage response (DDR), p53 activates SENP3, which activity enhances anti-tumor immune responses, a	Involved in cell cycle regulation as a trans-activator that acts to negatively regulate cell division by controlling a set of genes required for this process	Acts as a tumor suppressor in many tumor types; induces growth arrest or apoptosis depending on the physiological	ESCR, LFS	MDM2, BRCA1, PARP1

							mechanism that is facilitated by cGAS		circumstances and cell type		
ARID1A	O14497	242	N	-	-	UB	In cancers where ARID1A is deleted, there's an activation of the STING-mediated innate immune response	Involved in transcriptional activation and repression of select genes by chromatin remodeling (alteration of DNA-nucleosome topology)	tumors characterized by mutations or reduced expression of both ARID1A and ATM/Chk2 exhibited increased lymphocyte infiltration and were less aggressive		SMARCA4
KRAS	P01116	22	CM, Cytosol	Activated by a guanine nucleotide-exchange factor	Sotorasib, adagrasib	UB	For cancers with KRAS mutations, it is established that mtDNA release into the cyto-plasm can activate STING, and suppression of STING has been linked to immune evasion in lung cancer cells with KRAS mutations	Plays an important role in the regulation of cell proliferation	Plays a role in promoting oncogenic events by inducing transcriptional silencing of tumor suppressor genes in CRC cells in a ZNF304-dependent manner	AML, JMML, NS3, GASC, CFC2, OES, SFM	RAF1, EGFR

N – nuclear; C – cytoplasmic; ER – endoplasmic reticulum; G – Golgi complex; M – mitochondrion; CM – cell membrane; EM – endosome membrane; LM – lysosome membrane; UB – ubiquitous; SAVI – STING-associated vasculopathy, infantile-onset; MTDPS15 – Mitochondrial DNA depletion syndrome 15, hepatocerebral type; GLC1P – Glaucoma 1, open angle, P; FTDALS4 – Frontotemporal dementia and/or amyotrophic lateral sclerosis 4; IIAE8 – Encephalopathy, acute, infection-induced, 8, herpes-specific; AGS1 – Aicardi-Goutieres syndrome 1; SLE – Systemic lupus erythematosus; CHBL1 – Chilblain lupus 1; OPLL – Ossification of the posterior longitudinal ligament of the spine; GACI1 – Arterial calcification of infancy, generalized, 1; T2D – Type 2 diabetes mellitus; ARHR2 – Hypophosphatemic rickets, autosomal recessive, 2; COLED – Cole disease; BC – breast cancer; TNBC – triple negative breast cancer; UCC – uterine corpus carcinoma; MRMV2 – Mirror movements 2; FANCR – Fanconi anemia, complementation group R; MWS – Muckle-Wells syndrome; CINCA – Chronic infantile neurologic cutaneous and articular syndrome; KEFH – Keratoendothelitis fugax hereditaria; THC6 – Thrombocytopenia 6; WRS – Wolcott-Rallison syndrome; MEGAF – Megaloblastic anemia, folate-responsive; FTC – fallopian tube cancer; OC – ovarian cancer; BROVCA1 – Breast-ovarian cancer, familial, 1; PNCA 1–4 – Pancreatic cancer, type 1–4; BROVCA2 – Breast-ovarian cancer, familial, 2; GLM3 – Glioma 3; MDB – Medulloblastoma; PPC – primary peritoneal cancer; Pt – platinum based chemotherapy; ENDMC – Endometrial cancer; CRC – Colorectal cancer; AGM5 – Agammaglobulinemia 5, autosomal dominant; HIESI – Hyper-IgE recurrent infection syndrome 1, autosomal dominant; ADMIO1 – Autoimmune disease, multisystem, infantile-onset, 1; FANCV – Fanconi anemia, complementation group V; IMD83 – Immunodeficiency 83, susceptibility to viral infections; SCAN2 – Spinocerebellar ataxia, autosomal recessive, with axonal neuropathy 2; ALS4 – Amyotrophic lateral sclerosis 4; FANCD2 – Fanconi anemia complementation group D2; LFS – Li-Fraumeni syndrome; PC – Prostate cancer; OSRC – Osteogenic sarcoma; AISIMD – Autoinflammatory syndrome, familial, with or without immunodeficiency; MODY11 – Maturity-onset diabetes of the young 11; COFS4 – Cerebro-oculo-facio-skeletal syndrome 4; BDCHS – Budd-Chiari syndrome; PV – Polycythemia vera; ESCR – Esophageal cancer; AML – Leukemia, acute myelogenous; JMML – Leukemia, juvenile myelomonocytic; NS3 – Noonan syndrome 3; GASC – Gastric cancer; CFC2 –Cardiofaciocutaneous syndrome 2; OES –

Oculoectodermal syndrome; SFM – Schimmelpenning-Feuerstein-Mims syndrome; AT – Ataxia telangiectasia; MPLPF – Myeloproliferative/lymphoproliferative neoplasms, familial; NBSLD – Nijmegen breakage syndrome-like disorder; ATLD1 – Ataxia-telangiectasia-like disorder 1; IMD68 – Immunodeficiency 68; WM1 – Macroglobulinemia, Waldenstrom, 1; IIAE7 – Encephalopathy, acute, infection-induced, 7, herpes-specific; IMD39 – Immunodeficiency 39; SGMRT2 – Singleton-Merten syndrome 2;
